# Missing Americans: Early death in the United States—1933–2021

**DOI:** 10.1093/pnasnexus/pgad173

**Published:** 2023-05-29

**Authors:** Jacob Bor, Andrew C Stokes, Julia Raifman, Atheendar Venkataramani, Mary T Bassett, David Himmelstein, Steffie Woolhandler

**Affiliations:** Department of Global Health, Boston University School of Public Health, 801 Massachusetts Avenue, Boston, MA 02118, USA; Department of Global Health, Boston University School of Public Health, 801 Massachusetts Avenue, Boston, MA 02118, USA; Department of Health Law, Policy, and Management, Boston University School of Public Health, 715 Albany Street, Boston, MA 02118, USA; Leonard Davis Institute for Health Economics, University of Pennsylvania, 3641 Locust Walk, Philadelphia, PA 19104, USA; Medical Ethics and Health Policy, University of Pennsylvania, 423 Guardian Drive, Philadelphia, PA 19104, USA; François-Xavier Bagnoud (FXB) Center for Health and Human Rights, Harvard T.H. Chan School of Public Health, 677 Huntington Avenue, Boston, MA 02115, USA; Hunter College, City University of New York, 695 Park Avenue New York, NY 10065, USA; Cambridge Health Alliance, Harvard Medical School, 1493 Cambridge Street, Cambridge, MA 02139, USA; Hunter College, City University of New York, 695 Park Avenue New York, NY 10065, USA; Cambridge Health Alliance, Harvard Medical School, 1493 Cambridge Street, Cambridge, MA 02139, USA

**Keywords:** demography, excess deaths, United States, international comparisons, Human Mortality Database, racial disparities, COVID-19

## Abstract

We assessed how many US deaths would have been averted each year, 1933–2021, if US age-specific mortality rates had equaled the average of 21 other wealthy nations. We refer to these excess US deaths as “missing Americans.” The United States had lower mortality rates than peer countries in the 1930s–1950s and similar mortality in the 1960s and 1970s. Beginning in the 1980s, however, the United States began experiencing a steady increase in the number of missing Americans, reaching 622,534 in 2019 alone. Excess US deaths surged during the COVID-19 pandemic, reaching 1,009,467 in 2020 and 1,090,103 in 2021. Excess US mortality was particularly pronounced for persons under 65 years. In 2020 and 2021, half of all US deaths under 65 years and 90% of the increase in under-65 mortality from 2019 to 2021 would have been avoided if the United States had the mortality rates of its peers. In 2021, there were 26.4 million years of life lost due to excess US mortality relative to peer nations, and 49% of all missing Americans died before age 65. Black and Native Americans made up a disproportionate share of excess US deaths, although the majority of missing Americans were White.

**Significance Statement**

One million US deaths in 2020 and 1.1 million US deaths in 2021 would have been averted if the United States had the mortality rates of other wealthy nations. About half of these missing Americans died before age 65. The number of excess US deaths relative to peers is unprecedented in modern times, at least since the 1930s. These excess US deaths were a result of a decades-long divergence in mortality from other wealthy nations, beginning in the 1980s, and were further exacerbated by the COVID-19 pandemic. The use of an international benchmark highlights unfavorable mortality trends involving all US racial/ethnic groups and disproportionately affecting younger and working-age adults.

## Introduction

US residents died at higher rates during the COVID-19 pandemic than did residents of other wealthy nations (1, 2). This pattern of early death is not new (3–9). US life expectancy has been falling behind peer countries' since the 1980s and has stagnated in the last decade (10).

Excess mortality in the United States relative to other nations has been linked to proximate factors including drug overdoses, firearm injuries, vehicle accidents, cardiometabolic disorders, and smoking (3–7) and to fundamental causes including structural racism, economic inequality, gaps in access to medical care, and underinvestment in public health and social safety net programs in the United States (11). These factors have contributed to the large population-level impact of the COVID-19 pandemic in the United States as well as its disproportionate toll on people with lower income and education levels and Black, Hispanic, and Native American individuals (12, 13).

In this paper, we quantify the number of US deaths that would have been averted each year, from 1933 through 2021, if US age-specific mortality rates (ASMRs) had equaled the average of 21 other wealthy countries. We refer to these excess US deaths relative to peer countries as “missing Americans.” We note that “missing Americans” is a statistical construct; we cannot specify which deaths would have been averted, only the number that would have been averted if the United States had mortality rates of its peers.

Our use of an international benchmark—the average of other wealthy nations—to estimate “excess US deaths” differentiates our analysis from the large number of studies estimating “excess mortality” during the COVID-19 pandemic relative to prepandemic US death rates (14–18). By taking pre-COVID-19 death rates as “normal,” these approaches ignore longstanding mortality differences across nations and render invisible fundamental causes (19) of excess US mortality compared with other nations before and during the pandemic. Peer comparisons showcase the kind of mortality rates that are achievable in high-income countries and provide a better benchmark to judge where the United States stands. “Missing Americans” offers an easily interpretable metric to quantify the US mortality disadvantage and and augments the existing literature on life expectancy comparisons (20).

We extend prior analyses of excess US deaths in three directions (7, 8, 11, 21). First, we estimate long-term historical trends in excess US deaths, starting in 1933 (when the US mortality series begins). The longer view paints a clearer picture of the timing of the mortality divergence between the United States and other wealthy nations and variation in timing by age group. It also encompasses World War II (WWII), the largest mortality crisis prior to COVID-19 among today's wealthy nations (22).

Second, we examine the impact of COVID-19 on excess US deaths: the number of missing Americans in 2019, on the eve of the COVID-19 pandemic; the change in mortality in 2020 and 2021 associated with the COVID-19 pandemic in the United States and peer countries; and the impact of the pandemic on the number of missing Americans. We stratify our analyses by age, focusing in particular on adults under 65 years, who were already experiencing rising mortality prior to COVID-19 (5, 7, 8) and who are largely excluded from Social Security and Medicare and from social programs targeted to children.

Third, we use an international benchmark to assess trends in excess mortality borne by US racial and ethnic groups, given persistent racial disparities in life expectancy (23), rising mortality among White Americans (5), and differential mortality impacts of the pandemic (24, 25). Whereas studies of US health disparities typically focus on differences between US racial and ethnic groups, the declining health of White US residents has made it difficult to interpret trends in disparities. External comparisons shed light on the exceptional nature of US mortality trends and the experiences of different US racial and ethnic groups.

We conduct each of these analyses using recently released mortality data through 2021 for the United States and other wealthy nations, compiled by the Human Mortality Database (HMD) (26) and the US Centers for Disease Control and Prevention (CDC) (27).

## Methods

We compared mortality trends in the United States with mortality trends in 21 other wealthy countries. The comparison set of countries included Australia, Austria, Belgium, Canada, Denmark, Finland, France, Germany, Iceland, Ireland, Italy, Japan, Luxembourg, The Netherlands, New Zealand, Norway, Portugal, Spain, Sweden, Switzerland, and the United Kingdom. This comparison set included all countries with age-specific mortality data available from HMD beginning in 1960 or earlier and ending in 2020 or later, after excluding former-Soviet/Eastern Bloc nations. All countries included had 2021 gross domestic product (GDP) per capita >US$24,000 (28). Our comparison set makes comprehensive use of available data on peer countries, following recent work on mortality trends (1, 17, 29). However, we also present missing Americans estimates using two alternate comparison groups that have been used in international mortality comparisons: the other Group of Seven (G7) countries—Canada, France, Germany, Italy, Japan, and the United Kingdom—used in prior work by members of this study team (11) and the five largest Western European countries—France, Germany, Italy, Spain, and the United Kingdom—used by Preston and Vierboom (8) and Heuveline (21).

Data on deaths and population denominators starting in 1933 were obtained from the HMD, which compiles official data from national vital registries and censuses. We augmented the HMD standard long-term mortality series (available through 2019 to 2021 for the countries in the sample) with data from the HMD's Short-Term Mortality Fluctuations (STMF) database, which includes more complete data for 2020 and 2021. Both databases were accessed 2023 January 3; STMF was last updated 2022 December 17; HMD long series was last updated 2022 December 27. Details on the panel of countries are provided in Table S1. All countries were observed starting in 1933 with the exception of Portugal (1940), Austria (1947), Japan (1947), New Zealand (1948), Ireland (1950), Germany (1956), and Luxembourg (1960). The Germany series combines East and West Germany prior to 1989. All countries were observed through 2021, with the exception of Japan and Ireland, for which we carried forward 2020 ASMRs to 2021.

For each country, we extracted annual ASMRs, deaths, and denominators (exposure time) for 5-year age groups from the HMD long series. Seven countries had detailed data from the HMD long series through 2021. All remaining countries had data at least through 2019 (Table S1). For countries missing 2020 and/or 2021 data, we extracted data on deaths using the original input data from the STMF database. Countries reported deaths for different age bands in the STMF database, and we allocated deaths within STMF database age groups to 5-year age groups based on the age distribution of deaths in the last year available from the HMD long series. Denominators for 2020 and 2021 were obtained by fitting a linear trend to the last 5 years of exposure data available from the HMD long series (e.g. Italy, 2015–2019) for each 5-year age group in each country to obtain predictions for the most recent years. For country-years with overlapping data between the STMF database and HMD long series, ASMRs were very similar, supporting the validity of our approach (Table S1). Finally, we aggregated deaths and exposure time to the 10-year age bands reported by the United States in the STMF database: 0–4, 5–14, 15–24, …, and 85 + years. We also constructed six broader age groups to facilitate presentation of age-stratified analyses: 0–14, 15–44, 45–64, 65–74, 75–84, and 85 + .

As a benchmark against which to compute excess US mortality, we calculated the average ASMR across the 21 other wealthy countries for each age group and year (excluding countries with no data available for that year). We weighted countries by population [following (8)], comparing the mortality risks faced by US residents with a representative set of residents of other wealthy nations. (We present unweighted results in the supplement.) We then computed the number of deaths that would have occurred in the United States if the US population had experienced the age-specific mortality profile of peer nations. We subtracted this number from the actual number of US deaths at each age to compute the number of excess US deaths each year, i.e. the number of missing Americans.

We also calculated excess US years of life lost (YLL). YLL captures the number of years that a person would have been expected to live had they survived, placing greater weight on deaths at younger ages. We defined “excess US YLL” as the YLL that would be averted if the United States had the ASMRs of other wealthy nations. We estimated YLL associated with observed US deaths, YLL associated with US deaths under counterfactual (peer country) ASMRs, and “excess US YLL” computed as the difference between US observed and counterfactual YLL estimates. For each of these estimates, we weighted deaths by the age-specific life expectancy of other wealthy nations in the year the death occurred (details are provided in the supporting information).

Next, we compared ASMRs for US racial/ethnic groups relative to the average of other wealthy nations, combining all racial/ethnic groups in those countries into a single benchmark. We obtained data on US mortality and population denominators for 1999–2021, stratified by age and race/ethnicity, from the Multiple Cause of Death Files hosted on the CDC WONDER database (downloaded: 2022 January 5). We computed ASMRs for non-Hispanic Black (henceforth Black), non-Hispanic White (White), Hispanic, non-Hispanic Asian/Pacific Islander (Asian/Pacific Islander), and non-Hispanic American Indian/Alaskan Native (Native American) persons. Data from 2021 were provisional. Details are provided in the supporting information. For each racial/ethnic group, ASMRs were extracted for 10-year age bands, aligned with the data from the HMD. To avoid unstable estimates in race-by-age strata with low mortality rates, we aggregated to wider age bands: 0–14, 15–44, 45–64, 65–74, 75–84, and 85 + years. We computed mortality rate ratios and excess deaths for each US racial/ethnic group relative to the average of other wealthy nations. Replication data and code for all tables and figures have been posted in a public data repository (30).

## Results

### Long-term mortality trends in the United States and other wealthy nations prior to the COVID-19 pandemic

**Fig. 1.**
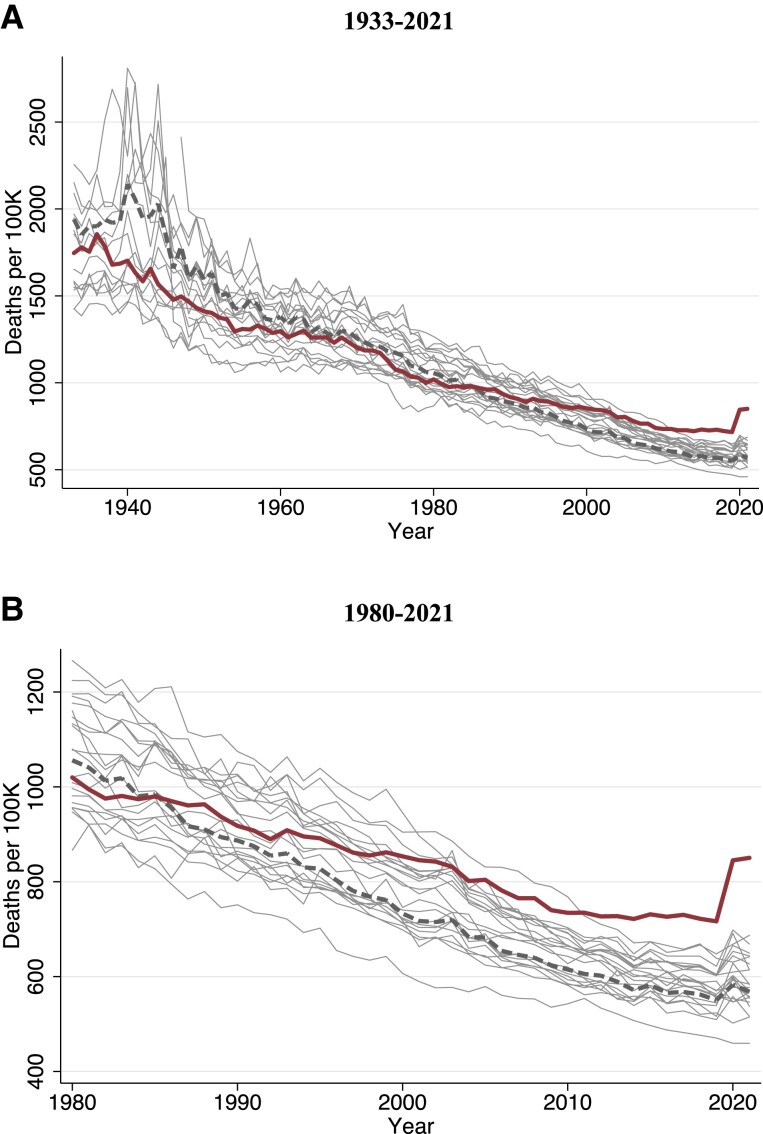
Age-standardized mortality trends in the United States and other wealthy nations. Figure shows deaths per 100,000 person-years: A) 1933–2021 and B) 1980–2021. The solid thick red line is the United States, the dashed thick grey line is the population-weighted average of 21 other wealthy nations, and the thin grey lines are country-specific trends for each of the other nations. Total mortality was age-standardized to the 2000 US population age distribution.

Figure 1 displays long-term trends, 1933–2021, in age-standardized mortality rates for each of the countries in the study. Mortality has fallen dramatically across all countries. The early time series bears the imprint of WWII and its aftermath, with high mortality rates observed in Europe and Japan. The United States had similar age-standardized mortality rates as other wealthy nations in the 1960s–1970s. However, since the 1980s, the position of the United States relative to other wealthy nations has progressively deteriorated.

Figure S1 stratifies these trends by age group, revealing substantial age heterogeneity in the US position relative to other countries. Despite similar aggregate mortality rates in the 1960s and 1970s, the United States had higher mortality at ages 15–64 years and lower mortality among people ages 75 years and older during this period. These patterns changed in the 1980s and 1990s as the mortality advantage of older US residents disappeared. During the period 2000–2021, the gap between the United States and other wealthy countries widened particularly among nonelderly adults (15–64 years). Mortality among US nonelderly adults slowed its decline after 2000 and increased in absolute terms starting in 2010 (Fig. S1).

The changing position of the United States relative to other wealthy nations is visualized in Fig. S2, which displays trends in age-specific mortality ratios between 1933 and 2021. The US mortality advantage in the late 1930s and 1940s reflects the disproportionate mortality shock of WWII on younger age groups in many of the comparison countries. Post-WWII, however, younger US age groups had elevated mortality rates relative to peer nations, while older US age groups had lower mortality. This pattern began to shift in the late 1970s as the US advantage at older ages started to diminish. Since 2000, trends in rate ratios for different age groups have diverged, increasing rapidly for persons under 65 years while remaining approximately stable (until the onset of the COVID-19 pandemic) for the older age group.

The COVID-19 pandemic appears as a spike in mortality in 2020 and 2021 (Fig. 1). The size of the 2020 increase differed by country and age group. While many countries experienced mortality increases among persons 75 years and older, the United States far outpaced other countries in the mortality among adults under 65 years (Fig. S1). From 2020 to 2021, mortality rates in the United States and most other peer nations declined in older age groups (over 75 years). However, in the United States, mortality continued to rise in younger age groups.

**Fig. 2.**
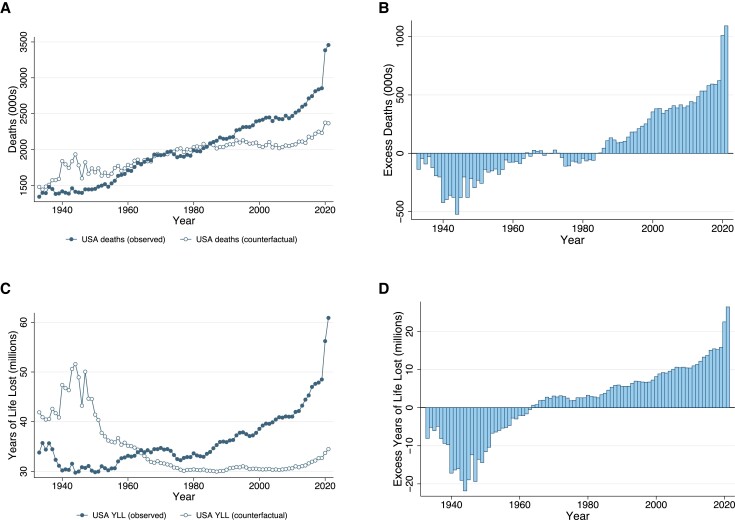
Excess deaths and YLL in the United States relative to other wealthy nations, 1933–2021. Source: Human Mortality Database. Note: The figure shows number of deaths in the United States and the counterfactual, the number of deaths that would have occurred in the United States if the United States had ASMRs equal to the average of 21 other wealthy nations. The average of other wealthy nations excludes Portugal prior to 1940, Austria and Japan prior to 1947, New Zealand before 1948, Ireland before 1950, Germany prior to 1956, and Luxembourg prior to 1960. From 1960, all countries are represented. The panels show: A) deaths in the United States and the counterfactual; B) the difference between the two, i.e. the number of excess US deaths or “missing Americans” each year; C) and D) analogous plots for YLL, where each death is weighted by the age-specific life expectancy of other wealthy nations in the year it occurred. Plotting points for all figures are provided in Table S3.

Figure 2A shows, for each year 1933–2021, the number of deaths that occurred in the United States and the counterfactual, the number of deaths that would have occurred if the United States had the ASMRs of other wealthy nations. Figure 2B shows the number of missing Americans each year. Since the early 1980s, the United States has experienced a steady and approximately linear increase in the number of excess deaths relative to other wealthy nations. In 2019 alone, on the eve of the COVID-19 pandemic, there were 622,534 excess US deaths (Table 1 and Fig. 2B). Between 1980 and 2019, 11.0 million excess US deaths occurred (Fig. 2 and Table S3).

**Table 1.** Excess US mortality relative to other wealthy nations: 2019–2021.

Year	Age	Population	Deaths per 100,000		Total deaths		Excess deaths		
			*USA*	*OWN*	*USA observed*	*Counterfactual*	*Ratio*	*Difference*	*% Excess*
2019	0–14 years	60,855,842	49	28	30,090	17,115	1.8	12,975	43%
	15–44 years	130,554,528	132	55	171,943	71,450	2.4	100,493	58%
	45–64 years	83,636,314	640	396	535,361	331,192	1.6	204,169	38%
	65–74 years	31,471,591	1,765	1,331	555,586	418,758	1.3	136,829	25%
	75–84 years	15,775,462	4,362	3,674	688,049	579,552	1.2	108,497	16%
	85 + years	6,200,544	14,092	13,131	873,791	814,220	1.1	59,572	7%
	*Total*	328,494,281	869	680	2,854,820	2,232,286	1.3	622,534	22%
2020	0–14 years	60,965,772	47	26	28,735	16,031	1.8	12,704	44%
	15–44 years	131,381,180	163	57	213,800	74,839	2.9	138,962	65%
	45–64 years	83,802,944	754	413	631,712	345,726	1.8	285,986	45%
	65–74 years	32,554,324	2,072	1,413	674,552	460,044	1.5	214,508	32%
	75–84 years	15,957,785	5,152	3,927	822,101	626,642	1.3	195,458	24%
	85 + years	6,101,087	16,601	13,948	1,012,825	850,976	1.2	161,849	16%
	*Total*	330,763,092	1,023	718	3,383,724	2,374,258	1.4	1,009,467	30%
2021	0–14 years	60,869,364	49	27	29,659	16,157	1.8	13,503	46%
	15–44 years	131,920,458	187	58	246,239	76,949	3.2	169,290	69%
	45–64 years	83,592,706	830	414	693,811	345,995	2.0	347,816	50%
	65–74 years	33,487,111	2,158	1,410	722,549	472,010	1.5	250,539	35%
	75–84 years	16,552,198	4,995	3,785	826,793	626,479	1.3	200,314	24%
	85 + years	6,210,527	15,080	13,331	936,553	827,912	1.1	108,641	12%
	*Total*	332,632,364	1,039	711	3,455,604	2,365,501	1.5	1,090,103	32%
Change, 2019–20	0–14 years		−2	−2	−1,354	−1,084	.	−270	.
	15–44 years		31	2	41,857	3,388	12.4	38,469	92%
	45–64 years		114	17	96,351	14,534	6.6	81,817	85%
	65–74 years		307	83	118,966	41,287	2.9	77,679	65%
	75–84 years		790	253	134,052	47,090	2.8	86,962	65%
	85 + years		2,509	817	139,034	36,757	3.8	102,277	74%
	*Total*		154	38	528,905	141,972	3.7	386,933	73%
Change, 2019–21	0–14 years		−1	−2	−431	−958	.	528	.
	15–44 years		55	4	74,296	5,499	13.5	68,797	93%
	45–64 years		190	18	158,450	14,803	10.7	143,647	91%
	65–74 years		392	79	166,963	53,253	3.1	113,710	68%
	75–84 years		634	111	138,744	46,927	3.0	91,817	66%
	85 + years		988	199	62,762	13,692	4.6	49,070	78%
	*Total*		170	32	600,784	133,215	4.5	467,569	78%

Source: Human Mortality Database. Note: Estimates for 2021 are based on provisional data. Other wealthy, population weighted average of 21 other wealthy nations; Ratio, observed/counterfactual US deaths; % Excess, ratio of excess US deaths to total deaths, expressed as a percentage.

The rising number of missing Americans was driven by a reduction in the US mortality advantage over 65 years in the 1980s and 1990s and increases in the US mortality disadvantage under 65 years since 2000 (Fig. S3). By 2019, half (51%) of these excess US deaths were in persons younger than 65 years. In 2019, 43% of all US deaths under 65 years and 14% of all US deaths over 65 years would have been avoided if US mortality rates had equaled the average of other wealthy nations (Tables 1 and S2).

The increase in excess US mortality is even more stark when using a YLL metric. Figure 2C shows YLL for observed deaths and the counterfactual. YLL increased in the United States after 1980; however, this increase would not have occurred if the United States had the ASMRs of other wealthy countries. Figure 2D shows the difference in YLL associated with excess US deaths. The gap in YLL between the United States and the counterfactual began to grow in the 1960s but has accelerated since the 1990s. Between 1980 and 2019, YLL attributable to excess deaths increased from 3.2 million per year to 15.8 million per year, claiming a cumulative total of 334.8 million YLL (Table S3). The rise in excess US deaths and excess US YLL was not explained by population aging, as shown in Fig. S4, which shows age-standardized trends.

**Fig. 3.**
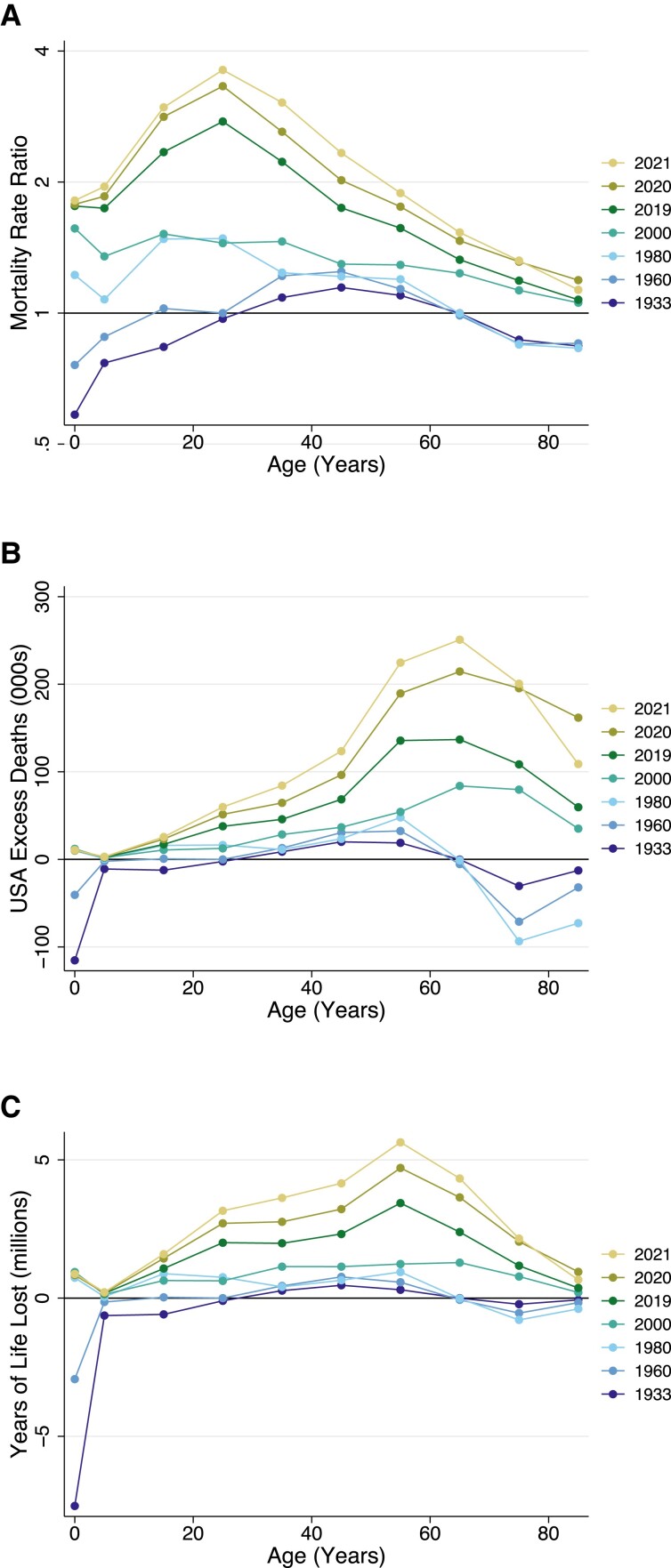
Excess US mortality by age group for select years: 1933–2021. Source: Human Mortality Database. Note: The figure shows A) mortality rate ratios, B) excess US deaths, and C) YLL in the United States relative to what would have occurred if the United States had ASMRs equal to the average of 21 other wealthy nations. The group of other wealthy nations excludes Portugal, Austria, Japan, New Zealand, Ireland, and Germany in 1933. From 1960, all countries are represented. Age groups are 0–4, 5–14, 15–24, …, and 85 + years.

Figure 3 presents detailed age profiles of US excess mortality for selected years: 1933, 1960, 1980, 2000, and 2019–2021. The figure displays mortality rate ratios, excess US deaths, and excess YLL in the United States relative to other wealthy nations. In all plots, higher numbers reflect worse outcomes for the United States relative to its peers. The divergence of the United States from peer countries occurred at different points in time for different age groups. As shown in Fig. 3A, in both 1933 and 1960, ASMRs were broadly similar in the United States and the average of peer countries. These years bookend a period, during WWII and its aftermath, when US mortality rates were substantially lower than those of peer nations. From 1960 to 1980, US-to-peer country mortality rate ratios increased for younger age groups (15–34 years). From 1980 to 2000, mortality rate ratios increased for older age groups (65 + years), although they remained lower than the mortality rate ratios for younger adults. From 2000 to 2019, US mortality rate ratios increased dramatically among young and middle-aged adults.

Figure 3B shows the number of excess US deaths by age and year. These absolute magnitudes are greater at older ages when most deaths occur. Figure 3C shows these differences using the YLL metric, which places additional weight on deaths occurring earlier in life. Figure 3B and C affirms that the absolute increase in excess US deaths and YLL between 1980 and 2000 occurred at older ages, while the increase in excess US deaths and YLL between 2000 and 2019 occurred primarily among non-elderly adults.

### Impact of COVID-19 pandemic on the US mortality disadvantage

The last 2 years of our mortality series reflect the toll of the COVID-19 pandemic, which in 2020 caused the largest single-year increase in mortality for the United States and many other wealthy countries since WWII. From 2019 to 2020 and 2021, mortality increased in all US age groups aged 15 and older (Fig. S1 and Table 1).

Other countries experienced smaller increases in AMSRs in 2020 and 2021 than did the United States. If the United States had the ASMRs of other wealthy nations, the increase in US deaths between 2019 and 2020 would have been 73% smaller, and the increase between 2019 and 2021 would have been 78% smaller. During this period, the number of missing Americans grew dramatically, from 622,534 in 2019 to 1,009,467 in 2020 and 1,090,103 in 2021. These excess US deaths accounted for 30% and 32%, respectively, of all US deaths (from all causes) in 2020 and 2021 (Tables 1 and S2).

The COVID-19 pandemic took a much larger toll on young and working-age adults in the United States than in other wealthy nations (Fig. S1). If the United States had the ASMRs of its peers, 88% of the 2019 to 2020 increase in US mortality under age 65 and 92% of the 2019 to 2021 increase in US mortality under age 65 would have been avoided. Excess US deaths accounted for 50% of all US deaths under age 65 (from all causes) in 2020 and 55% of all US deaths under age 65 in 2021 (Tables 1and S2). During 2020 and 2021, there were 968,261 excess US deaths under 65 years, nearly half of the total number of missing Americans during this period.

Annual YLL associated with the US mortality disadvantage increased from 15.8 million in 2019 to 22.5 million in 2020 and 26.4 million in 2021, highlighting both the greater increase in mortality in the United States relative to peer countries and the younger ages at which these deaths occurred (Fig. 2 and Table S3). Estimates of excess US deaths in 2019–2021 were qualitatively similar when using the alternative international comparison sets of the G7 and five western European countries and when using the unweighted average of peer nations (Table S4). Between 1980 and 2021, there were a total of 13.1 million excess US deaths and 383.7 million excess YLL.

### Who are the missing Americans? Excess mortality by US race/ethnicity

**Fig. 4.**
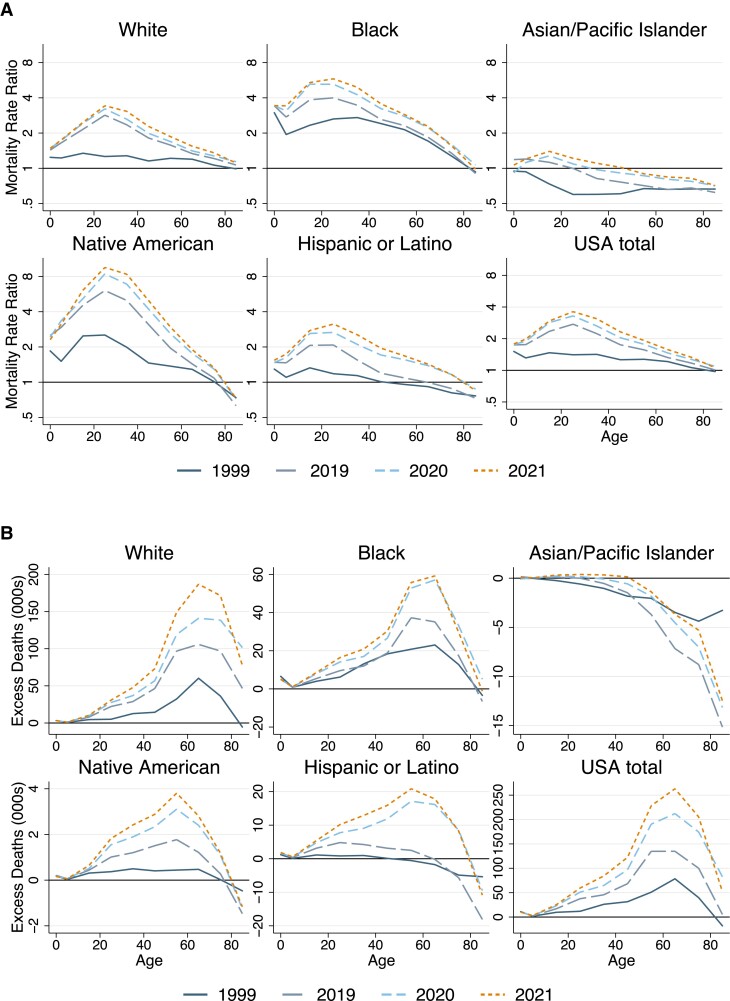
Excess mortality of US racial/ethnic groups relative to the average of other wealthy nations, 1999–2021. Sources: Multiple Cause Mortality Files from CDC Wonder and Human Mortality Database. The figure shows A) mortality rate ratios comparing US racial/ethnic groups with the average of 21 other wealthy nations and B) numbers of excess US deaths for each group, stratified by age: 0–14, 15–44, 45–64, 65–74, 75–84, and 85 + years. US racial/ethnic groups are Hispanic, non-Hispanic Black (Black), non-Hispanic White (White), non-Hispanic Asian/Pacific Islander (Asian/Pacific Islander), and non-Hispanic American Indian/Alaskan Native (Native American).

Figure 4A presents ratios comparing ASMRs for each US racial/ethnic group with the average of other wealthy countries. Data are presented for 4 years—1999, 2019, 2020, and 2021—enabling assessment of trends in excess US deaths by race/ethnicity both before and during the COVID-19 pandemic. The figure shows important commonalities across different US racial/ethnic groups. First, mortality rate ratios relative to other nations increased over time for all race/ethnicities, indicating worsening position relative to other wealthy nations. Second, mortality rate ratios were largest among younger adults, indicating that the pattern of elevated early- and mid-life US mortality is not limited to one specific race or ethnicity.

However, there were also key differences in levels and trends (Fig. 4, Table 2). In 2019, Black and Native American US residents aged 15–44 years had mortality rates 3.8 and 5.3 times higher, respectively, than the average of other wealthy countries. Mortality among White and Hispanic US residents ages 15–44 years was also elevated at 2.5 and 1.8 times the average of other nations, respectively. In contrast, in 2019 Asian/Pacific Islander US residents had mortality rates below the average of other nations. White and Native Americans experienced the largest relative increases in mortality from 2000 to 2019. Figure 4B displays the age profile of excess deaths by year and race/ethnicity, revealing how differences in the population size and age distribution shape racial/ethnic differences in excess deaths.

**Table 2.** Excess US deaths by race/ethnicity relative to other wealthy nations: 2019–2021.

Race/ethnicity	Age	Population (2019)	2019			2020			2021		
			Deaths	Excess	%	Deaths	Excess	%	Deaths	Excess	%
White	0–14	31,524,362	13,076	4,191	32%	12,528	4,347	35%	12,661	4,388	35%
	15–44	72,844,178	99,126	58,933	59%	115,919	74,095	64%	131,410	88,684	67%
	45–64	55,142,376	367,261	143,243	39%	405,621	175,911	43%	451,591	222,341	49%
	65–74	23,716,452	421,407	105,838	25%	485,693	140,994	29%	530,676	186,863	35%
	75–84	12,436,703	553,760	96,866	17%	639,678	138,502	22%	654,536	171,484	26%
	85+	5,241,403	734,885	46,614	6%	831,544	101,492	12%	775,270	77,520	10%
	Total	200,905,474	2,189,515	455,685	21%	2,490,983	635,342	26%	2,556,143	751,280	29%
Black	0–14	9,227,154	8,615	6,001	70%	8,387	5,897	70%	8,637	6,119	71%
	15–44	18,703,220	36,962	26,993	73%	49,524	39,083	79%	56,512	45,844	81%
	45–64	10,248,785	96,151	56,171	58%	121,202	79,562	66%	127,687	86,137	67%
	65–74	3,149,591	77,055	35,147	46%	103,700	57,088	55%	105,734	59,243	56%
	75–84	1,387,735	68,321	17,339	25%	89,555	33,183	37%	84,003	29,670	35%
	85+	519,318	61,638	−6,556	−11%	79,412	5,102	6%	69,858	−1,164	−2%
	Total	43,235,803	348,742	135,095	39%	451,780	219,916	49%	452,432	225,849	50%
Asian/PI	0–14	3,553,388	1,180	188	16%	995	−49	−5%	1,152	96	8%
	15–44	9,495,354	4,940	−435	−9%	5,911	288	5%	6,777	1,034	15%
	45–64	5,076,497	13,881	−5,181	−37%	17,700	−2,411	−14%	18,813	−1,250	−7%
	65–74	1,582,888	13,899	−7,163	−52%	18,889	−4,531	−24%	19,689	−3,671	−19%
	75–84	747,503	18,673	−8,788	−47%	23,982	−7,018	−29%	24,585	−5,294	−22%
	85+	304,936	24,899	−15,143	−61%	32,096	−13,154	−41%	30,781	−12,466	−40%
	Total	20,760,566	77,472	−36,523	−47%	99,573	−26,875	−27%	101,796	−21,552	−21%
AIAN	0–14	604,145	439	269	61%	392	242	62%	375	223	59%
	15–44	1,177,885	3,271	2,648	81%	4,600	3,948	86%	5,541	4,875	88%
	45–64	650,645	5,855	3,278	56%	8,117	5,442	67%	9,356	6,687	71%
	65–74	212,383	4,041	1,215	30%	5,551	2,399	43%	5,954	2,811	47%
	75–84	90,180	3,589	276	8%	4,842	1,095	23%	4,791	1,180	25%
	85+	30,205	2,501	−1,465	−59%	3,253	−1,258	−39%	3,146	−1,165	−37%
	Total	2,765,443	19,696	6,222	32%	26,755	11,869	44%	29,164	14,610	50%
Hispanic	0–14	15,661,797	6,560	2,117	32%	6,220	2,079	33%	6,604	2,417	37%
	15–44	28,066,338	27,252	12,154	45%	37,364	21,555	58%	44,597	28,443	64%
	45–64	12,205,136	50,104	5,609	11%	76,551	29,001	38%	84,191	36,759	44%
	65–74	2,822,119	37,337	−214	−1%	58,239	16,147	28%	59,784	17,800	30%
	75–84	1,307,751	42,375	−5,669	−13%	62,231	8,493	14%	60,039	8,244	14%
	85+	509,096	48,759	−18,093	−37%	65,092	−9,497	−15%	60,445	−10,844	−18%
	Total	60,572,237	212,387	−4,095	−2%	305,697	67,778	22%	315,660	82,819	26%

Source: Public-use Multiple Cause of Death Files from CDC Wonder and the Human Mortality Database. Note: Estimates for 2021 are based on provisional data. Table displays rates for US racial/ethnic groups: Hispanic, non-Hispanic White (White), non-Hispanic Black (Black), non-Hispanic Asian/Pacific Islander (Asian), and non-Hispanic American Indian/Alaskan Native (AIAN).

**Fig. 5.**
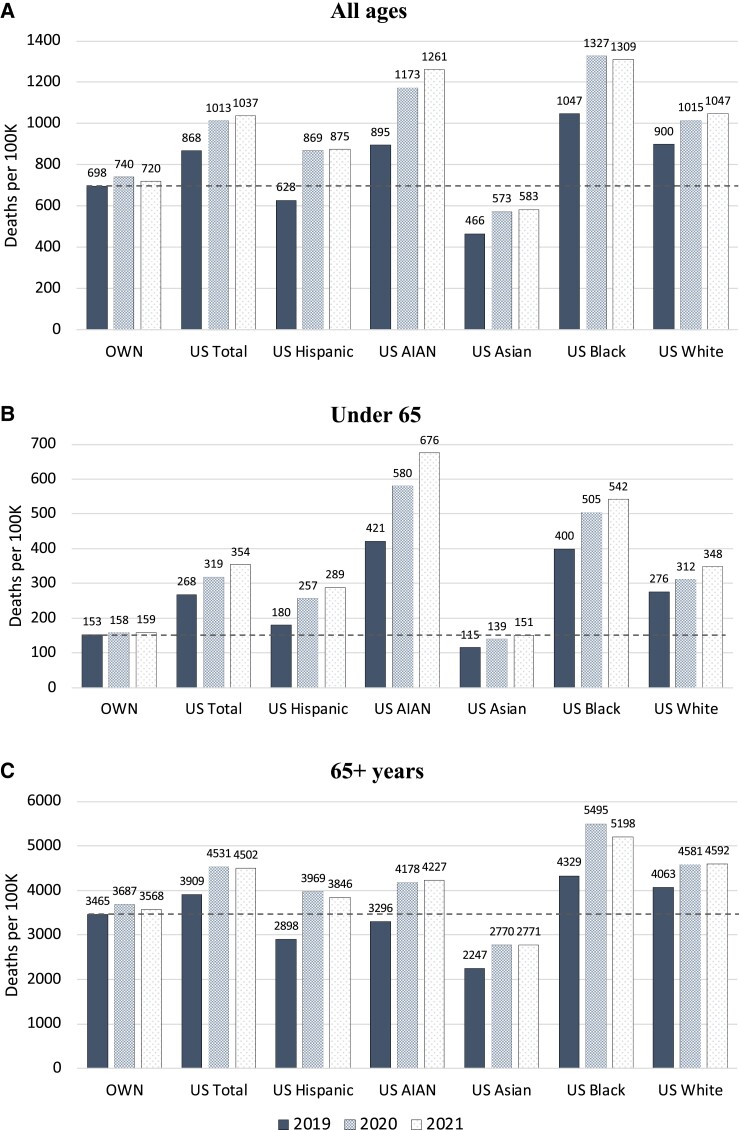
Age-standardized mortality rates for US racial and ethnic groups and the average of other wealthy nations: 2019, 2020, and 2021. Source: CDC Wonder and Human Mortality Database. Notes: OWN, other wealthy nations. Figure shows age-standardized mortality rates for A) all ages, B) under 65 years, and C) 65 + years. Mortality rates are standardized to the US population age distribution in each year. Mortality rates are shown for the following US populations: Hispanic, non-Hispanic White (White), non-Hispanic Black (Black), non-Hispanic Asian/Pacific Islander (Asian), and non-Hispanic American Indian/Alaskan Native (AIAN). Dashed line shows 2019 (pre-COVID) mortality rates for the average of other wealthy nations.

During the COVID-19 pandemic, mortality rates rose for all US racial/ethnic groups (Fig. 5). Native American, Black, and Hispanic US residents experienced the largest increases, and nonelderly adults of these racial and ethnic groups experienced the largest relative increases compared with other wealthy nations. For example, from 2019 to 2021, age-standardized, under-65 mortality increased by just 6 deaths per 100,000 in other wealthy nations. However, in the United States, under-65 mortality increased by 254 deaths per 100,000 among Native American residents, 143 deaths per 100,000 among Black residents, 108 deaths per 100,000 among Hispanic residents, 72 deaths per 100,000 among White residents, and 36 deaths per 100,000 among Asian residents (Fig. 5). The increase in Hispanic Americans' death rates during the pandemic largely erased their previous survival advantage relative to non-Hispanic White Americans and pushed their mortality rates above the average of other wealthy countries at all but the oldest ages. By 2021, Black and Native Americans ages 15-44 had mortality rates 5.3 and 8.3 times higher than the average of other wealthy nations; White and Hispanic Americans ages 15–44 had mortality rates 3.0 and 2.7 times the peer country average (Fig. 4A, Table 2).

The data reveal noteworthy changes between 2020 and 2021. Whereas mortality rates declined among Black Americans in the second year of the pandemic, mortality continued to increase among White and Native Americans (Fig. 5A), leading to large increases in excess United States deaths in these populations. From 2020 to 2021, all racial/ethnic groups experienced a substantial increase in mortality rates among persons under 65 years (Fig. 5B). This occurred amidst falling mortality at older ages among US Black and Hispanic residents and little change in over-65 mortality for other racial/ethnic groups (Fig. 5C). White US adults over 65 saw no reduction in mortality between 2020 and 2021.

Table 2 and Fig. 4B show the absolute number of missing Americans by US racial/ethnic group. Despite higher mortality rates among Black and Native Americans, in absolute numbers, the majority of missing Americans were White, reflecting the much larger size and older age distribution of the US White population. While White Americans accounted for 61% of the population and experienced 69% of excess deaths in 2021, Black Americans accounted for 13% of the population and 21% of excess deaths. Hispanic Americans, who are younger on average, made up 19% of the population and 8% of excess deaths (Table 2).

## Discussion

The current US mortality disadvantage is unprecedented in modern times: there were more “missing Americans” in 2021 than at any other point going back to 1933. The United States had lower death rates than other wealthy nations in the 1930s through 1950s and similar death rates in the 1960s and 1970s. US death rates began to diverge in the 1980s, and this divergence has accelerated in the 21st century. When COVID-19 emerged, Americans were already dying at much higher rates than residents of other wealthy nations. There were 622,534 excess US deaths in 2019 alone and 11.0 million total excess US deaths between 1980 and 2019. The annual death toll from the US mortality disadvantage increased to 1,009,467 in 2020 and 1,090,103 in 2021, reflecting the catastrophic loss of life the United States experienced during the pandemic, far in excess of other wealthy nations. Missing Americans represent a large share of under-65 mortality in the US. In 2020 and 2021, more than half of all US deaths under 65 years would have been averted if the United States had experienced the mortality rates of its peers. During the first two years of the COVID-19 pandemic, there were nearly 1 million excess US deaths under 65 years. The 1.1 million missing Americans in 2021 would have enjoyed an estimated 26.4 million additional years of life had they survived.

Our findings are consistent with recent reports indicating a growing life expectancy gap between the United States and other countries (17, 22, 29, 31, 32). Using a similar set of comparator countries, Woolf and colleagues estimated that the US life expectancy deficit increased from 1.9 years to 3.1 years between 2010 and 2018 and widened further to 4.7 years in 2020 (17) and 5.3 years in 2021 (29). While period life expectancy is a widely used summary measure of mortality, it may be misinterpreted as reflecting mortality differences at end of life rather than at younger ages where the greatest differences lie between the United States and peer nations.

“Missing Americans” offers an alternative, easily interpretable metric: the number of US deaths that would have been averted if the United States had the ASMRs of its peers. Our analysis builds on our (11) and others' (7, 8) previous analyses documenting excess US deaths prior to COVID-19 and during the pandemic (21), as well as research on the racial distribution of excess deaths before the pandemic (11, 33) and in pandemic-associated mortality (24, 25, 34, 35).

Other studies have computed “excess deaths” relative to different benchmarks. Our choice of an international comparator offers insights not available from comparison groups within the United States. For example, studies computing excess mortality relative to pre-COVID-19 trends within the same country take prepandemic mortality as given (14). However, US mortality has diverged from other wealthy nations since the 1980s, after tracking close to other nations for most of the post-WWII period. As we show, there were over 600,000 missing Americans in 2019 even before COVID-19. High levels of excess US mortality are likely to continue even as COVID mortality wanes. Other studies have compared mortality rates across racial/ethnic or socioeconomic groups within the United States (24). However, life expectancy of White Americans—particularly those with less than a college degree and those residing in nonmetro areas—has stagnated or fallen since the 1990s (5, 36). Using White Americans as the reference group or benchmark for comparison renders invisible the mortality crisis among White US residents and underestimates excess mortality for other US racial and ethnic groups.

About half of the missing Americans died before reaching age 65. The United States has had higher working-age mortality than other wealthy nations since the 1960s, but the gap has widened dramatically in recent years. Excess US deaths under 65 doubled between 2000 and 2019 and then increased by an additional two-thirds between 2019 and 2021. During the pandemic, half of all US deaths under 65 years—and 90% of the increase in under-65 mortality since 2019—would have been averted if the United States had the ASMRs of its peers.

Reports by the National Academies of Science, Engineering, and Medicine have identified multiple proximate causes for excess US mortality at working ages (3, 7). Higher rates of homicides, motor vehicle fatalities, sexually transmitted infections including HIV/AIDS, drug-related mortality, obesity and diabetes, heart disease, and lung disease explained much of the US mortality disadvantage during the 1990s and early 2000s (3). From 2000 to 2019, the increase in excess US mortality under 65 years was due largely to increases in drug overdoses, alcohol-related mortality, suicides, and cardiometabolic diseases (7). In addition to having shorter lives, US working-age adults experience greater morbidity than do residents of peer countries. The World Health Organization estimates than in 2019, US “health-adjusted life expectancy” (HALE) lagged unadjusted life expectancy by 12.4 years—more life-years spent in poor health than any other wealthy nation (37). Higher prevalence of diabetes and obesity in the United States likely contributed to elevated COVID-19 mortality among working-age adults, due to increased severity of COVID-19 infection (38). Deaths to working-age adults have social impacts not directly captured in mortality statistics, including psychological trauma and economic precarity for surviving children, friends, and family, as well as lost economic productivity and social and political participation (39–42).

The large spike in early death in the United States during the 2020–2021 COVID-19 pandemic was due not only to the emergence of a novel pathogen. Other nations were similarly exposed. Rather, the mortality impact of the pandemic in the United States was a product of longstanding social inequities and policy failures that made the United States particularly vulnerable, factors that had also contributed to the US mortality disadvantage before the pandemic. US policies throughout the country's history have harmed the health of Black and Native Americans (23), beginning with the genocide of native populations and the enslavement of people of African descent (43, 44) and continuing through long periods of legal segregation, political disenfranchisement, and economic exclusion. These harms have reverberated through the intergenerational transfer of resources, power, and health.

In recent decades, policy failures have adversely affected the health of US residents of all racial/ethnic groups, particularly those without a college degree (45). Since the 1970s, the United States—like other wealthy countries—has undergone structural economic changes, with increased exposure to trade (46), automation (47), and a shift to service sector employment. The United States has failed to protect less-educated workers from the adverse health consequences of these changes. Stagnant minimum wages and losses of collective bargaining protections (48) have contributed to widening economic inequality. A scant safety net for working-age adults and the absence of universal healthcare have privatized risk, tying health more closely to personal wealth and employment (49). Additionally, lax regulation of opioids, firearms, environmental pollutants, unhealthy foods, and workplace safety has contributed to elevated US mortality, particularly among lower-educated and lower-income people. Increasingly divergent policies at the state level have resulted in widening health gaps across US states (50). In those geographic areas of the United States where excess mortality has increased the most, voters have turned towards policy-makers who have further undermined population health (51, 52), e.g. through refusal to expand Medicaid or to implement firearm regulations (53). Underlying these policy trends, the commitment of a majority of US White residents to a political program of deregulation and divestment from the social determinants of health has hamstrung government efforts to protect the health of all US residents, inflicting the greatest harm on the most vulnerable, incuding Americans of color and White Americans without a college degree (54).

During COVID-19, these same “fundamental causes”—socioeconomic inequality, a limited safety net, and a politics unaccountable to population health—manifested in the large increase in excess US deaths that we report here. Residential segregation, limited ability to work from home, distrust of the medical establishment, decreased access to health care, and a higher prevalence of comorbid conditions contributed to excess risk among low-income and minoritized populations (34, 55). Gaps in the safety net forced many low-income people to work despite high infection risk (13). Housing insecurity increased crowding and transmission at home. The exclusion of immigrant populations from the safety net (56), anemic workplace protections (57), and crowded housing (58) contributed to the large impact of COVID-19, particularly among US Hispanic residents (59). Failure to mandate that employers give their workers time off to get vaccinated contributed to disparities in uptake (60). Finally, the politicization of the pandemic—including mask use, physical distancing, and vaccine uptake—has resulted in substantial COVID-19–related mortality and contagion, particularly among US White residents and those with less than a college education (61). The rise in mortality among White US residents between 2020 and 2021 was likely a consequence of low vaccine uptake in this population. We note that the rise in US mortality during the pandemic included a large rise in deaths due to drug overdoses (62), reflecting the failure of US policy to adequately address its longstanding drug crisis.

In contrast to the decentralized approach to pandemic control taken in the United States, other countries launched rapid, coordinated national responses; had greater adherence to control measures; outpaced the United States in vaccine uptake; had better baseline health conditions due to universal health care and a stronger safety net; coordinated economic policy that sought to maintain ties between employers and their workers; and avoided long-term interruptions in children's schooling that occurred in the United States. These countries not only weathered the emergency phase of the pandemic better than the United States but were also better poised to protect future population health at the close of 2021.

Our analysis has limitations. First, the mortality data for 2021 are preliminary due to the lag time between the occurrence and registration of deaths. Still, in previous years, by 26 weeks after the end of the year, 99% of US deaths have been recorded (63). Data for Japan and Ireland had not yet been posted for 2021 and were imputed using 2020 death rates. Second, differential reporting of US race and ethnicity on death certificates and in Census denominators may bias estimates. In particular, American Indian/Alaskan Native “race” and Hispanic “ethnicity” are underreported on death certificates, which may lead to underestimation of deaths for these groups and overestimation for other groups (64, 65). Third, the literature on US mortality in international context has used a range of country comparison sets and results vary somewhat depending on the countries included and whether weighted or unweighted averages are used. We utilized data on all other wealthy countries with a lengthy time series, excluding former communist countries, which experienced a large mortality crisis in the 1990s following the collapse of the Soviet Union. However, our findings are qualitatively similar when using alternate comparison sets used in other studies of excess US deaths (8, 11) or using unweighted averages of peer countries. Due to data limitations, the composition of our comparison group changed between 1933 and 1960; however, it was consistent after 1960. Fourth, our analysis takes a period approach to estimating the number of missing Americans, similar to the literature on international life expectancy comparisons. Estimating the number of US residents who would be alive today if counterfactual mortality rates persisted for their lifetime would require a different, cohort-based approach. Finally, the missing Americans in this analysis do not reflect all the US residents lost to premature death—nor do they reflect all deaths that would be averted if the US had the mortality rates of international top performers.

The future of COVID-19 mortality is uncertain, but even a full return to prepandemic conditions would hardly end the US mortality crisis. Our study showed that even before the pandemic, the United States experienced more than 600,000 excess US deaths each year, with most occurring among Americans under 65 years. How can the United States reduce the number of missing Americans? Expanding current US policies with demonstrated health impact and ensuring that all US residents have access to health care and public social welfare benefits when they need them are places to start. For example, research has documented the health benefits of expanded access to Medicaid (66, 67), minimum wage and Earned Income Tax Credit laws (68), unemployment insurance subsidies (69), nutritional assistance (70), early childhood education (71), and firearm (72) and environmental regulations (73). Learning from peer nations, the United States could extend social protections and universal health coverage to nonelderly adults and enhance the regulation of pollutants and firearms. Preventing future missing Americans will require efforts to address fundamental causes of poor population health in the United States that existed even before the pandemic began (44, 74).

## Supplementary Material

pgad173_Supplementary_Data

## Data Availability

Source data are freely available from public repositories (CDC WONDER and Human Mortality Database) with a request to use the data: https://wonder.cdc.gov/mcd.html, https://mortality.org/. The replication code and analytic datasets for this paper have been posted to the OSF repository, https://osf.io/bawu6/, DOI: 10.17605/OSF.IO/BAWU6.
