# Supplementary material for: Missing Americans: Early death in the United States—1933–2021
Source: PNAS Nexus. 2023 May 29;2(6):pgad173. doi: 10.1093/pnasnexus/pgad173 (PMC10257439; doi:10.1093/pnasnexus/pgad173)
Supplement: pgad173_Supplementary_Data [file pgad173_supplementary_data.docx]

Missing Americans: Early Death in the United States, 1933-2021

Jacob Bor^1*^, Andrew C. Stokes^1^, Julia Raifman^2^, Atheendar Venkataramani^3,4^, Mary T. Bassett^5^, David Himmelstein^6,7^, Steffie Woolhandler^6,7^

**SUPPLEMENTARY MATERIALS**

**Contents:**

Appendix A. Materials and Methods

Appendix B. Supplementary Figures (Figs. S1 to S4)

Appendix C. Supplementary Tables (Tables S1 to S4)

Appendix A. Materials and Methods

Materials

*Data sources*

Data on deaths and population denominators were obtained from the Human Mortality Database (HMD),(25) which compiles official data from national vital registries and censuses. HMD is maintained by researchers at the Max Planck Institute for Demographic Research and the University of California-Berkeley.

The standard HMD mortality “long series” includes annual, age-specific mortality data (deaths, exposure time, and rates) for 41 countries, starting as early as 1751 (Sweden) and extending up to 2020 (for most countries) and to 2021 for seven countries in the sample. These data are harmonized into a standardized format. During the COVID-19 pandemic, due to increased demand for real-time data, HMD compiled and published provisional mortality data released by national vital statistics agencies. These data, published as the Short-Term Mortality Fluctuations (STMF) database include weekly death counts for 38 countries from the mid-2010s (for most countries) through mid-2022. The STMF data are updated regularly. A limitation of the STMF database is that different countries report different age bands for provisional deaths. While HMD publishes harmonized age categories, these categories are very broad (e.g. 15-65 years is a single group). Instead, we utilized the original input data provided by countries that forms the basis for the STMF database.

The STMF database has limitations. Data may be incomplete due to lags between the date of death and registration, although such issues are mitigated as the STMF database is updated. Data from the U.K. are reported based on date of registration rather than date of occurrence. Data from Canada exclude deaths in the Yukon (<0.1% of Canada's population). Finally, Japan and Ireland were not currently included in the STMF database, although their HMD “long series” data both run through 2020.

While a pre-print version of this paper presented data as of June 2022 (<https://www.medrxiv.org/content/10.1101/2022.06.29.22277065v2.full.pdf>), there were some updates to the HMD database during peer review enabling the inclusion of additional countries and more accurate data. To ensure that we were using the most complete data available, we reran the analysis and updated the paper with data accessed January 3, 2023. The STMF data used had been last updated December 17, 2022; HMD long series data had been last updated December 27, 2022. Results were not substantially different from what were reported in the pre-print.

*Countries and country-years included in the analysis*

We compared mortality trends in the U.S. with mortality trends in 21 other wealthy countries (OWN). The comparison set of countries included: Australia, Austria, Belgium, Canada, Denmark, Finland, France, Germany, Iceland, Ireland, Italy, Japan, Luxembourg, Netherlands, New Zealand, Norway, Portugal, Spain, Sweden, Switzerland, and United Kingdom. These countries represented all countries with mortality data available from HMD beginning in 1960 or earlier and ending in 2020 or later, after excluding former-Soviet/Eastern Bloc nations. Former-Soviet/Eastern Bloc nations were excluded because they experienced very different political, economic, and demographic trends than the other nations included in the study. Many former Soviet states experienced a major increase in mortality during the 1990s following the fall of the Soviet Union, an experienced not shared by the U.S. and the other comparator countries.

Details on the panel of countries are provided in **Table S1**. Our analysis includes all country-years from 1933, the first year of U.S. data, through 2021. All countries were observed starting in 1933 with the exception Portugal (1940), Austria (1947), Japan (1947), New Zealand (1948), Ireland (1950), Germany (1956), and Luxembourg (1960). The Germany series combines East and West Germany prior to 1989. Japan and Ireland were not included in the STMF database and long-series HMD data were only available through 2020. Therefore, we carried forward Japan’s and Ireland’s 2020 age-specific mortality rates to 2021. All other countries were observed through 2021.

*Data extraction and manipulation*

For each country, we extracted annual age-specific mortality rates (ASMR), deaths, and denominators (exposure time) for 5-year age groups from the HMD long series. We then extracted data on deaths for the most recent years using the original input data from the STMF database. We aggregated the STMF weekly data to annual counts. Because different countries reported deaths for different age bands in the STMF database, we allocated deaths within STMF database age groups to 5-year age groups based on the distribution of deaths in the last year available from the HMD long series. For example, in the STMF database, Germany reports deaths for 5-year age groups among persons 30 and over but collapses all ages under 30 years. Using the distribution of deaths in 2020 – Germany’s last year in the “long series” – we reallocated deaths under 30 years to ages 0-4, 5-9, …, 25-29 for 2021. Denominators to match the STMF death counts were obtained by fitting a linear trend to the 2016-2020 exposure data available from the HMD long series for each 5-year age group in each country (e.g. Germany, ages 40-44, 2016-2020) to obtain predictions for 2021 (e.g. Germany, ages 40-44, 2021).

Our final dataset consisted of annual age-specific mortality rates by 5-year age group, for 22 countries (U.S. and 21 “other wealthy nations”) with data from 1933 through 2021. We assessed the accuracy of the STMF data by comparing the STMF and HMD estimates for country-years with overlapping data between the two datasets. ASMRs were very similar **(Table S1).** Finally, to avoid false precision in reporting, we aggregated deaths and exposure time to the ten-year age bands reported by the U.S. in the STMF data: 0-4, 5-14, 15-24, …, 85+ years. Additionally, for our age-stratified analyses, we constructed six wider age groups to facilitate visual and tabular presentation of the data: 0-14, 15-44, 45-64, 65-74, 75-84, 85+.

As a benchmark against which to compute “excess” U.S. mortality, we calculated the average ASMR across “other wealthy nations” for each age group and year (excluding countries whose data were not available for that year). We weighted countries by population (following (8)), and compared the mortality rates of U.S. residents to the weighted average mortality rates of residents of other wealthy nations. We note that some other studies have used unweighted averages of mortality rates in peer nations. The population-weighted approach is appropriate if, as in our study, we seek to understand the average death rate of people living in other wealthy nations. For completeness, we conducted the unweighted analysis as a robustness check. Data are reported in **Table S4**.

*Mortality data by U.S. race/ethnicity*

We calculated ASMRs for U.S. racial and ethnic population groups for 1999-2021 based on mortality figures and midyear population denominators from the CDC WONDER database.(26) Data were extracted for 1999-2020 from the “Current Final Multiple Cause of Death Data” files and separately for 2020-2021 from the “Provisional Multiple Cause of Death Data” files. (1999 was the earliest year available for the Multiple Cause of Death files on CDC WONDER.) Data were downloaded January 5^th^, 2023.

The “Current Final” data (1999-2020) report four “bridged race” categories: White, Black, Asian or Pacific Islander, and American Indian or Alaskan Native. To create these racial categories, CDC imputed a single race for deaths and denominators with multiple races for purposes of tabulation. The “Provisional” data (2020-2021) were obtained with six “single race” categories: White, Black, Asian, Pacific Islander, American Indian or Alaskan Native, and “more than one race”. We combined Asian and Pacific Islander so that the category was comparable with the bridged race categories. The presence of the “multiple race” category meant that death rates in the provisional data may be slightly biased. To address this limitation, we constructed correction factors for each race/ethnicity and age group equal to the ratio of the 2020 “final” mortality rate (computed on bridged race categories) to the 2020 “provisional” mortality rate (computed on single-race categories). This correction factor can be interpreted as capturing the extent to which people identifying with multiple races are assigned to different bridge race categories. We multiplied 2021 provisional mortality rates by this correction factor to estimate 2021 mortality for bridged race categories. We carried forward the 2020 population denominators to 2021 for the bridged race categories. We then estimated deaths in 2021 by multiplying these population denominators by the corrected mortality rates for each race and age group.

Using these data, we computed ASMRs for: Non-Hispanic Black (henceforth Black), Non-Hispanic White (White), Hispanic, Non-Hispanic Asian/Pacific Islander (Asian/Pacific Islander), and Non-Hispanic American Indian/Alaskan Native (Native American). For each population subgroup, ASMRs were computed for ten-year age groups, aligned with the data from HMD. For ease of exposition, we report on the wider age bands described above.

Methods

*Excess U.S. deaths*

For each year, 1933-2021, we compared U.S. ASMRs for each age group (0-14, 15-44, 45-64, 65-74, 75-84, 85+) with the ASMRs of people residing in 21 “other wealthy nations” (OWN). To assess excess mortality on a relative scale, we computed ASMR ratios for each year, 1933-2020, comparing the U.S. with the OWN benchmark. We additionally computed ratios comparing ASMRs for U.S. racial and ethnic population groups with the ASMRs of OWN, each year 1999-2021.

To assess excess mortality on an absolute scale, we calculated the number of deaths that would have been observed in the U.S. in each year if the U.S. had the ASMRs of the OWN (multiplying the U.S. population distribution by OWN ASMRs). We then subtracted this number from the number of observed deaths in the U.S. to compute “excess deaths”.

To compare changes in mortality associated with the COVID-19 pandemic, we computed changes in age-specific mortality rates from 2019 to 2020 and from 2019 to 2021 for the total U.S. population, for U.S. racial and ethnic groups, and for the comparison group of OWN.

*Years of life lost*

We also computed “years of life lost” (YLL) associated with excess U.S. deaths. YLL weights deaths by the number of years that a person would have been expected to live had they survived. A reasonable question is what life expectancy values to use for YLL calculations. If the U.S. had the ASMRs of its peers, not only would a large share of U.S. deaths be averted; but those people, had they survived, would have enjoyed more years of life, on average, due to lower mortality rates at older ages. Their life expectancy, in this counterfactual state of the world, is therefore a function of the mortality rates of OWN not of the U.S. In calculating YLL, we therefore chose to weight each death by the age-specific life expectancy of OWN in the year the death occurred. (Results are similar, if more difficult to interpret, when weighting by U.S. age-specific life expectancy.)

To compute observed U.S. YLL, counterfactual U.S. YLL, and excess U.S. YLL, we multiplied observed U.S. deaths, counterfactual U.S. deaths, and excess U.S. deaths by age- and year-specific life expectancy estimates for OWN and the U.S.. We constructed separate life tables for each year, 1933-2021, for the population-weighted average of other wealthy nations based on 5-year age-specific mortality rates (m_x_) from the HMD long series, augmented by the STMF database in recent years (0-4, 5-9, ..., 90+). For each country-year, we extracted values of “a_x_”, i.e. the number of years lived within an age interval by someone who dies in that interval, from HMD long-series life tables. We carried forward values of a_x_ when not available for 2020 and 2021. We calculated the average “a_x_” across other wealthy nations for each year and age interval. For the open interval (90+ years) we assumed a constant mortality hazard, such that a_90_ = 1/m_90_. Based on values of m_x_ and a_x_ for each age group, we estimated annual 5-year life tables for OWN and the U.S. using standard techniques.

Whereas 5-year life tables estimate age-specific life expectancy (e_x_) at the left-side of each age interval, for YLL calculations we are interested in the average life expectancy of persons who died at different ages across each age interval (i.e., at ages 15-19, not at age 15). We estimated average life expectancy of someone who died within the interval as: the average number of years of life lost in that interval among decedents (5-a_x_) plus the life expectancy of someone who survived to the end of the interval (e_x+5_). For the open interval (90+ years), we assumed life expectancy was e_90_ = 1/m_90_ for any age over 90. To facilitate YLL calculations, we aggregated our 5-year estimates of "average life expectancy of someone who died" to 10-year age groups used throughout the rest of the paper, weighting by the number of deaths in each interval.

**Appendix B. Supplementary Figures**

**(A) 1933-2021**

**(B) 1980-2021**

**(C) 2000-2021**

**Fig. S1. Age-specific mortality trends in the U.S. and other wealthy nations.** Source: Human Mortality Database. Note: Figure shows deaths per 100K population, (A) 1933-2021, (B) 1980-2021, and (C) 2000-2021. Dark red line is U.S.; dashed thick grey line is population-weighted average of other wealthy nations; solid thin grey lines are trends for other individual countries.

Fig. S2. Ratio of U.S. mortality to mortality in other wealthy nations, by age group. Note: Figure shows mortality rate ratios for each age group, 1933-2021. The average of other wealthy nations excludes Portugal prior to 1940, Austria and Japan prior to 1947, New Zealand prior to 1948, Ireland prior to 1950, Germany prior to 1956, and Luxembourg prior to 1960. From 1960, all 21 countries are represented.

(A) Ages 0-64 years

(B) Age 65 years and older

**Fig. S3. Missing Americans, 1933-2021, under 65 years vs. 65 and older.** Note: For each age range, figure shows difference between observed deaths in the U.S. and the number of deaths that would have occurred in the U.S. if the U.S. had age-specific mortality rates equal to the average of 21 other wealthy nations. The average of other wealthy nations excludes Portugal prior to 1940, Austria and Japan prior to 1947, New Zealand prior to 1948, Ireland prior to 1950, Germany prior to 1956, and Luxembourg prior to 1960. From 1960, all countries are represented.**(A) Excess U.S. deaths, adjusted for changes in age distribution**

**(B) Years of life lost, adjusted for changes in age distribution**

**Fig. S4. Excess U.S. deaths and years of life lost, 1933-2021, standardized by U.S. 2000 age distribution.** Note: Figure (A) shows the difference between observed deaths in the U.S. and the number of deaths that would have occurred in the U.S. if the U.S. had age-specific mortality rates equal to the average of 21 other wealthy nations. Figure (B) shows the similar plot for years of life lost. In each plot, the solid line shows the same data as Fig 2. The dashed line shows estimates standardized to the 2000 U.S. age distribution. The average of other wealthy nations excludes Portugal prior to 1940, Austria and Japan prior to 1947, New Zealand prior to 1948, Ireland prior to 1950, Germany prior to 1956, and Luxembourg prior to 1960. From 1960, all countries are represented.

**Appendix C. Supplementary Tables**

**Table S1. Panel of countries included in the analysis**

| **Country** | **Country Code** | **Start year (HMD long)** | **End year**  **(HMD long)** | **End year (STMF)** | **% Diff: HMD long vs. STMF** | **Population (2020)** |
| --- | --- | --- | --- | --- | --- | --- |
|  |  |  |  |  |  |  |
| Australia | AUS | 1933 | 2020 | 2021 | 0.1% | 25,990,250 |
| Austria | AUT | 1947 | 2019 | 2021 | -2.3% | 9,010,743 |
| Belgium | BEL | 1933 | 2021 | 2021 | 0.1% | 11,568,653 |
| Canada | CAN | 1933 | 2020 | 2021 | -0.1% | 38,426,762 |
| Switzerland | CHE | 1933 | 2021 | 2021 | 0.0% | 8,703,647 |
| Denmark | DNK | 1933 | 2021 | 2021 | 0.2% | 5,856,866 |
| Spain | ESP | 1933 | 2020 | 2021 | 0.1% | 47,568,022 |
| Finland | FIN | 1933 | 2021 | 2021 | 0.2% | 5,541,473 |
| France | FRA | 1933 | 2020 | 2021 | 0.2% | 65,587,348 |
| Germany | GER | 1956 | 2020 | 2021 | 0.2% | 83,457,710 |
| Ireland | IRL | 1950 | 2020 | N/A | N/A | 4,982,855 |
| Iceland | ISL | 1933 | 2020 | 2021 | 0.1% | 375,469 |
| Italy | ITA | 1933 | 2019 | 2021 | 1.2% | 59,487,834 |
| Japan | JPN | 1947 | 2020 | N/A | N/A | 123,423,636 |
| Luxembourg | LUX | 1960 | 2020 | 2021 | 0.2% | 642,874 |
| Netherlands | NLD | 1933 | 2019 | 2021 | -0.3% | 17,540,875 |
| Norway | NOR | 1933 | 2020 | 2021 | 0.1% | 5,418,375 |
| New Zealand | NZL | 1948 | 2021 | 2021 | 0.1% | 5,113,692 |
| Portugal | PRT | 1940 | 2021 | 2021 | 0.3% | 10,332,833 |
| Sweden | SWE | 1933 | 2021 | 2021 | -0.6% | 10,417,555 |
| United Kingdom | UK | 1933 | 2020 | 2021 | 0.0% | 67,173,059 |
| United States | USA | 1933 | 2020 | 2021 | 0.4% | 332,632,365 |
|  |  |  |  |  |  |  |
| Note: Demographic data were obtained from the Human Mortality Database (HMD). We extracted data both from the HMD long series (HMD long) and from the HMD short term mortality fluctuations dataset (STMF). We included all countries represented in the HMD that had data starting in 1960 or earlier and extending to 2020 or later in the HMD, as of December 27, 2022. We excluded former Soviet and Eastern Bloc countries (with the exception of East Germany, which was combined with West Germany pre-1989). Percent difference (% diff) between HMD long and STMF reports the percent difference in total number of deaths in the two databases for those country-years where both were represented. STMF data were not used for those countries with HMD long series data through 2021. Japan and Ireland had HMD long series through 2020 but did not have STMF data; we carried forward 2020 data to 2021. | | | | | | |

**Table S2. Excess mortality in the U.S. relative to other wealthy nations: 2019-2021**

(Note: Table replicates Table 1, with data aggregated to ages 0-64 and 65+ years)

**Note:** percent excess is defined as the ratio of excess U.S. deaths to total U.S. deaths, expressed as a percentage. When reporting changes 2019 to 2020 or 2019 to 2021, percent excess is the ratio of the change in excess U.S. deaths to the change in total U.S. deaths.

**Table S3. Excess U.S. deaths and years of life lost (plotting points Figure 2)**

|  |  | **Deaths (000s)** | | |  | **Years of Life Lost (millions)** | | |
| --- | --- | --- | --- | --- | --- | --- | --- | --- |
| **Year** |  | **USA** | **OWN** | **Excess** |  | **USA** | **OWN** | **Excess** |
| 1933 |  | 1,342,112 | 1,479,349 | -137,237 |  | 33.8 | 41.9 | -8.1 |
| 1934 |  | 1,396,915 | 1,441,964 | -45,049 |  | 35.7 | 40.9 | -5.2 |
| 1935 |  | 1,392,747 | 1,483,685 | -90,938 |  | 34.4 | 40.4 | -6.0 |
| 1936 |  | 1,479,244 | 1,508,156 | -28,912 |  | 35.7 | 40.5 | -4.9 |
| 1937 |  | 1,450,438 | 1,572,669 | -122,231 |  | 34.5 | 42.6 | -8.1 |
| 1938 |  | 1,381,384 | 1,573,626 | -192,242 |  | 32.3 | 41.7 | -9.4 |
| 1939 |  | 1,387,871 | 1,590,412 | -202,541 |  | 31.1 | 40.9 | -9.7 |
| 1940 |  | 1,417,271 | 1,838,727 | -421,456 |  | 30.2 | 47.4 | -17.2 |
| 1941 |  | 1,397,632 | 1,793,456 | -395,824 |  | 30.4 | 46.8 | -16.3 |
| 1942 |  | 1,385,195 | 1,744,216 | -359,021 |  | 30.3 | 46.3 | -16.0 |
| 1943 |  | 1,459,544 | 1,836,162 | -376,618 |  | 31.5 | 50.6 | -19.1 |
| 1944 |  | 1,411,336 | 1,933,062 | -521,726 |  | 29.8 | 51.6 | -21.8 |
| 1945 |  | 1,401,719 | 1,780,433 | -378,714 |  | 30.0 | 48.9 | -18.9 |
| 1946 |  | 1,395,628 | 1,598,044 | -202,416 |  | 30.8 | 43.2 | -12.4 |
| 1947 |  | 1,445,377 | 1,822,873 | -377,496 |  | 30.7 | 50.1 | -19.4 |
| 1948 |  | 1,444,345 | 1,661,300 | -216,955 |  | 31.1 | 44.6 | -13.6 |
| 1949 |  | 1,443,612 | 1,737,974 | -294,362 |  | 30.2 | 44.6 | -14.4 |
| 1950 |  | 1,452,459 | 1,682,716 | -230,257 |  | 29.8 | 41.4 | -11.6 |
| 1951 |  | 1,482,117 | 1,738,917 | -256,800 |  | 30.0 | 40.4 | -10.4 |
| 1952 |  | 1,496,829 | 1,634,854 | -138,025 |  | 31.1 | 37.8 | -6.7 |
| 1953 |  | 1,517,543 | 1,678,506 | -160,963 |  | 30.7 | 37.1 | -6.4 |
| 1954 |  | 1,481,067 | 1,631,401 | -150,334 |  | 30.2 | 36.2 | -6.0 |
| 1955 |  | 1,528,710 | 1,663,098 | -134,388 |  | 30.6 | 36.0 | -5.4 |
| 1956 |  | 1,564,470 | 1,744,112 | -179,642 |  | 30.6 | 35.8 | -5.2 |
| 1957 |  | 1,633,143 | 1,772,019 | -138,876 |  | 32.0 | 36.7 | -4.7 |
| 1958 |  | 1,646,068 | 1,703,067 | -56,999 |  | 32.6 | 35.4 | -2.7 |
| 1959 |  | 1,660,180 | 1,737,022 | -76,842 |  | 32.8 | 35.8 | -3.0 |
| 1960 |  | 1,711,981 | 1,787,164 | -75,183 |  | 33.2 | 35.2 | -2.0 |
| 1961 |  | 1,701,515 | 1,771,972 | -70,457 |  | 33.0 | 35.1 | -2.1 |
| 1962 |  | 1,756,713 | 1,844,064 | -87,351 |  | 33.1 | 34.8 | -1.6 |
| 1963 |  | 1,813,544 | 1,858,885 | -45,341 |  | 33.9 | 34.4 | -0.5 |
| 1964 |  | 1,798,040 | 1,792,922 | 5,118 |  | 34.3 | 33.7 | 0.6 |
| 1965 |  | 1,828,136 | 1,853,150 | -25,014 |  | 33.9 | 33.1 | 0.8 |
| 1966 |  | 1,863,149 | 1,836,580 | 26,569 |  | 34.3 | 32.4 | 1.8 |
| 1967 |  | 1,851,304 | 1,834,252 | 17,052 |  | 33.9 | 31.9 | 1.9 |
| 1968 |  | 1,930,088 | 1,907,711 | 22,377 |  | 34.5 | 31.7 | 2.7 |
| 1969 |  | 1,921,987 | 1,940,157 | -18,170 |  | 34.5 | 32.1 | 2.4 |
| 1970 |  | 1,921,006 | 1,921,215 | -209 |  | 34.7 | 31.7 | 3.0 |
| 1971 |  | 1,927,512 | 1,929,564 | -2,052 |  | 34.4 | 31.6 | 2.8 |
| 1972 |  | 1,963,934 | 1,935,452 | 28,482 |  | 34.5 | 31.4 | 3.1 |
| 1973 |  | 1,972,994 | 1,970,822 | 2,172 |  | 34.1 | 31.2 | 2.9 |
| 1974 |  | 1,934,414 | 1,970,703 | -36,289 |  | 33.3 | 30.8 | 2.5 |
| 1975 |  | 1,892,879 | 2,002,711 | -109,832 |  | 32.5 | 30.7 | 1.8 |
| 1976 |  | 1,909,414 | 2,016,742 | -107,328 |  | 32.3 | 30.4 | 1.9 |
| 1977 |  | 1,899,571 | 1,966,096 | -66,525 |  | 32.7 | 30.1 | 2.6 |
| 1978 |  | 1,927,801 | 2,002,299 | -74,498 |  | 32.9 | 30.3 | 2.6 |
| 1979 |  | 1,913,877 | 1,997,487 | -83,610 |  | 32.9 | 30.3 | 2.6 |
| 1980 |  | 1,989,836 | 2,031,945 | -42,109 |  | 33.6 | 30.4 | 3.2 |
| 1981 |  | 1,977,962 | 2,043,294 | -65,332 |  | 33.2 | 30.3 | 2.9 |
| 1982 |  | 1,974,796 | 2,030,534 | -55,738 |  | 33.1 | 30.3 | 2.8 |
| 1983 |  | 2,019,193 | 2,079,550 | -60,357 |  | 32.9 | 30.4 | 2.6 |
| 1984 |  | 2,039,361 | 2,038,696 | 665 |  | 33.5 | 30.2 | 3.4 |
| 1985 |  | 2,086,445 | 2,082,369 | 4,076 |  | 33.9 | 30.1 | 3.8 |
| 1986 |  | 2,105,379 | 2,061,014 | 44,365 |  | 34.7 | 30.1 | 4.6 |
| 1987 |  | 2,123,316 | 2,015,115 | 108,201 |  | 35.3 | 30.0 | 5.4 |
| 1988 |  | 2,168,003 | 2,035,383 | 132,620 |  | 35.9 | 30.1 | 5.8 |
| 1989 |  | 2,150,458 | 2,035,544 | 114,914 |  | 36.0 | 30.1 | 5.9 |
| 1990 |  | 2,148,467 | 2,058,807 | 89,660 |  | 35.9 | 30.4 | 5.6 |
| 1991 |  | 2,169,513 | 2,073,841 | 95,672 |  | 36.2 | 30.6 | 5.6 |
| 1992 |  | 2,175,607 | 2,072,740 | 102,867 |  | 36.3 | 30.7 | 5.6 |
| 1993 |  | 2,268,550 | 2,129,003 | 139,547 |  | 37.3 | 30.9 | 6.4 |
| 1994 |  | 2,279,013 | 2,096,645 | 182,368 |  | 37.8 | 30.8 | 7.0 |
| 1995 |  | 2,312,122 | 2,128,917 | 183,205 |  | 37.9 | 31.0 | 6.8 |
| 1996 |  | 2,314,728 | 2,103,369 | 211,359 |  | 37.5 | 30.8 | 6.7 |
| 1997 |  | 2,314,215 | 2,081,385 | 232,830 |  | 37.1 | 30.5 | 6.6 |
| 1998 |  | 2,337,263 | 2,085,237 | 252,026 |  | 37.3 | 30.5 | 6.7 |
| 1999 |  | 2,391,392 | 2,096,947 | 294,445 |  | 37.8 | 30.5 | 7.2 |
| 2000 |  | 2,403,398 | 2,048,537 | 354,861 |  | 38.6 | 30.4 | 8.1 |
| 2001 |  | 2,416,399 | 2,035,115 | 381,284 |  | 39.2 | 30.4 | 8.8 |
| 2002 |  | 2,443,367 | 2,059,548 | 383,820 |  | 39.6 | 30.4 | 9.2 |
| 2003 |  | 2,448,274 | 2,104,889 | 343,385 |  | 39.6 | 30.5 | 9.1 |
| 2004 |  | 2,397,634 | 2,029,601 | 368,033 |  | 39.9 | 30.3 | 9.6 |
| 2005 |  | 2,448,015 | 2,063,341 | 384,675 |  | 40.5 | 30.4 | 10.1 |
| 2006 |  | 2,426,244 | 2,018,969 | 407,276 |  | 40.9 | 30.3 | 10.6 |
| 2007 |  | 2,423,735 | 2,034,880 | 388,855 |  | 40.8 | 30.4 | 10.4 |
| 2008 |  | 2,471,973 | 2,056,650 | 415,323 |  | 41.1 | 30.5 | 10.6 |
| 2009 |  | 2,437,113 | 2,047,368 | 389,745 |  | 41.0 | 30.6 | 10.4 |
| 2010 |  | 2,468,425 | 2,062,439 | 405,986 |  | 41.0 | 30.6 | 10.4 |
| 2011 |  | 2,515,442 | 2,072,105 | 443,337 |  | 42.0 | 31.0 | 11.0 |
| 2012 |  | 2,543,302 | 2,110,710 | 432,592 |  | 42.2 | 30.8 | 11.3 |
| 2013 |  | 2,596,961 | 2,112,144 | 484,817 |  | 43.2 | 31.1 | 12.2 |
| 2014 |  | 2,626,466 | 2,092,944 | 533,522 |  | 44.4 | 31.2 | 13.2 |
| 2015 |  | 2,712,594 | 2,179,230 | 533,364 |  | 45.3 | 31.6 | 13.7 |
| 2016 |  | 2,744,241 | 2,164,357 | 579,884 |  | 47.0 | 32.0 | 15.1 |
| 2017 |  | 2,813,541 | 2,220,515 | 593,026 |  | 47.6 | 32.2 | 15.4 |
| 2018 |  | 2,839,248 | 2,248,873 | 590,376 |  | 47.9 | 32.7 | 15.2 |
| 2019 |  | 2,854,820 | 2,232,286 | 622,534 |  | 48.5 | 32.7 | 15.8 |
| 2020 |  | 3,383,724 | 2,374,258 | 1,009,466 |  | 56.2 | 33.7 | 22.5 |
| 2021 |  | 3,457,018 | 2,365,501 | 1,091,517 |  | 60.9 | 34.5 | 26.4 |

**Table S4. Excess U.S. deaths relative to alternative international comparators: 2019-2021**

**Note:** first three columns are population-weighted averages of other wealthy nations. Last column is the simple (unweighted) average of other nations.
